# Characteristics of Single vs. Multiple Suicide Attempters Among Adult Population: A Systematic Review and Meta‑Analysis

**DOI:** 10.1192/j.eurpsy.2024.1641

**Published:** 2024-08-27

**Authors:** S. Abascal-Peiró, J. Herrera-Sánchez, L. Izaguirre-Gamir, J. Aznar-Carboné, A. Alacreu-Crespo, A. Porras-Segovia

**Affiliations:** ^1^Department of Psychiatry, Hospital Universitario Fundación Jiménez Díaz, Madrid; ^2^Department of Psychology and Sociology, Area of Personality, Assessment and Psychological Treatment, Universidad of Zaragoza, Teruel; ^3^Instituto de Investigación Sanitaria Fundación Jiménez Díaz, Madrid, Spain; ^4^Division of Psychiatry, Imperial College, London, United Kingdom

## Abstract

**Introduction:**

Suicide is one of the leading causes of unnatural death worldwide. There might be meaningful differences between those individuals that attempt suicide once in their lifespan and those who make multiple attempts in terms of sociodemographic and clinical characteristics. There are no previous meta-analysis addressing this topic in the adult population.

**Objectives:**

We aimed to examine the factors that differentiate single and multiple suicide attempters in adult population.

**Methods:**

We followed the Preferred Reporting Items for Systematic reviews and Meta-Analyses (PRISMA) guidelines to conduct this review and meta-analysis. The review protocol was registered in PROSPERO. We carried out a systematic literature search in three databases to identify original studies that explored the differences between single and multiple suicide attempters among adult population. A total of 75 studies were included in the review and 69 were included in the meta-analysis.

**Results:**

Multiple attempters were more likely to present certain disorders such as mood and psychotic disorders, as well as personality or substance use disorders. Higher suicide ideation and suicide intent scores also characterized this group. Childhood trauma experiences, stressful life events, and higher rates of hopelessness were statistically significant in multiple attempters.

**Conclusions:**

Identifying the factors predicting multiple suicide attempts helps to delineate a high-risk suicidal profile that should be taken into account in the clinical and suicide prevention scenario.

**Disclosure of Interest:**

None Declared

